# Genetic polymorphism and transcriptional regulation of *CREBBP* gene in patient with diffuse large B-cell lymphoma

**DOI:** 10.1042/BSR20191162

**Published:** 2019-08-13

**Authors:** Haifeng Zhao, Yutian Kan, Xinyuan Wang, Leiyuan Chen, Peng Ge, Zhengzi Qian

**Affiliations:** 1 Department of Hematology and Oncology, Tianjin Medical University Cancer Institute and Hospital, National Clinical Research Center for Cancer, Key Laboratory of Cancer Prevention and Therapy, Tianjin Clinical Research Center for Cancer, Tianjin, P.R. China; 2 Department of Laboratory, Tianjin Medical University Cancer Institute and Hospital, National Clinical Research Center for Cancer, Key Laboratory of Cancer Prevention and Therapy, Tianjin Clinical Research Center for Cancer, Tianjin, P.R. China; 3 Department of Lymphoma, Tianjin Medical University Cancer Institute and Hospital, National Clinical Research Center for Cancer, Key Laboratory of Cancer Prevention and Therapy, Tianjin Clinical Research Center for Cancer, Tianjin, P.R. China

**Keywords:** CREBBP, DLBCL, expression, polymorphism

## Abstract

In the present study, we aim to examine the relationship between genetic polymorphism and transcriptional expression of cyclic AMP response element binding protein (*CREBBP*) and the risk of diffuse large B-cell lymphoma (DLBCL). Two hundred and fifty healthy individuals and 248 DLBCL patients participated in the present study. The *CREBBP* rs3025684 polymorphism was detected by polymerase chain reaction-restriction fragment length polymorphism (PCR-RFLP). The mRNA expression of *CREBBP* was tested by the real-time quantitative PCR (RT-qPCR). The allele A frequency of *CREBBP* rs3025684 in DLBCL patients was obviously higher than that of controls (*P*=0.01). No significant difference was detected between *CREBBP* rs3025684 polymorphism and clinical characteristics of DLBCL patients when subgrouped according to different parameters. The results demonstrated that the allele A of *CREBBP* rs3025684 increased the susceptibility to DLBCL (*P*=0.004), with a worse overall survival (OS) rate (*P*=0.002), a worse progression-free survival (PFS) rate (*P*=0.033) and poor prognosis (*P*=0.003) in DLCBL patients. Furthermore, the expression of *CREBBP* mRNA was considerably decreased in DLBCL patients as compared with controls (*P*<0.001), and the expression in patients with GG genotype was up-regulated in comparison with patients with GA and AA genotype (*P*=0.016 and *P*=0.001, respectively). However, no statistical differences were found in OS (*P*=0.201) and PFS (*P*=0.353) between the lower *CREBBP* mRNA level subgroup and higher *CREBBP* mRNA level subgroup. These data suggested that the *CREBBP* gene may be an important prognostic factor in DLBCL patients and perform an essential function in the development of DLBCL.

## Introduction

Diffuse large B-cell lymphoma (DLBCL) is an aggressive non-Hodgkin lymphoma with extreme heterogeneity, accounting for 30–40% of newly diagnosed lymphomas [1]. Although the standard R-CHOP regimen has extremely good therapeutic effect on DLBCL patients, approximately 30–40% of patients show relapse and 10% have refractory disease [2]. In the past decades, accumulating evidences have shown the genetic, microenvironment, autoimmune diseases and occupational exposure participated in the pathogenesis of DLBCL [3–5]. With gene-expression profiling and next-generation sequencing, some common genetic loci are found to enmesh in the lymphomagenesis of DLBCL [6]. However, the pathogenesis of DLBCL is still not fully understood.

Single nucleotide polymorphism (SNP) is the most abundant type of genetic variation that occurs at a specific position in the human genome. Accumulating evidence indicated that SNPs have shown the ability to influence the activity and expression of the genes, and affect the pathogenesis and risk of an extensive range of cancer [7–10]. Previous studies have demonstrated that SNPs in *TP53, XRCC1* and *A20* are related to the risk of DLBCL [11–13]. Genome-wide association studies have recognized genetic susceptibility locus for DLBCL [14]. The contribution of SNP in histone-modifying enzymes to DLBCL pathogenesis is a research hotspot.

Somatic mutations in cyclic AMP response element binding protein (*CREBBP*) and *EP300*, and removal or inactivation of the HAT coding domain affect approximately 39% of DLBCL patients [15], and are also associated with Rubinstein Tyabi Syndrome (RTS) [16,17]. CREBBP belongs to the KAT3 family of histone/protein lysine acetyltransferases, which is a highly conserved and universally expressed nuclear phosphoprotein [18,19]. *CREBBP* inactivation expedites GC-derived pathogenesis of lymphoma [20]. Down-regulation of CREBBP is related to worse overall survival (OS) rate in pediatric acute lymphoblastic leukemia and may affect the response to chemotherapy [21]. *Crebbp*^+/−^ mice had blemish in the development of B-cell lymphoid and an increased incidence of hematopoietic malignancy [22]. The rs3025684 (A/G) is an SNP of *CREBBP* located in intron 21. This SNP was shown to be a possible risk factor for developing autism in the Netherlands, U.K. and Denmark, as well as among Bengali-Hindus [23,24].

However, the status of *CREBBP* rs3025684 SNP and the gene expression in DLBCL in a Chinese Han population is not completely understood. It was hypothesized that *CREBBP* rs3025684 polymorphism and expression may be related to the susceptibility and pathogenesis of DLBCL, and the results found that it may be a prognostic factor for DLBCL patients.

## Materials and methods

### Subjects

The present study recruited 250 healthy individuals and 248 DLBCL patients, diagnosed according to the World Health Organization classification [25] at Tianjin Medical University Cancer Institute and Hospital from 2011 to 2013. Peripheral blood and lymphoid specimens were collected before initial therapy. All participants signed the informed consent, and the approval of the study was obtained from the Tianjin Cancer Institute Institutional Review Board. The clinical and pathological characteristics of the patients and controls are presented in Table 1.

**Table 1:** Clinical characteristics of DLBCL patients

Characteristics	Number (*n*=248) *n* (%)	Control (*n*=250) *n* (%)
**Gender**		
Male	112 (45.16)	109 (43.60)
Female	136 (54.84)	141 (56.40)
**Age**		
≤60	165 (66.53)	184 (73.60)
>60	83 (33.47)	66 (26.40)
**Subtype**		
GCB	75 (30.24)	
nGCB	173 (69.76)	
**Ann-arbor stage**		
I–II	152 (61.29)	
III–IV	96 (38.71)	
**IPI score**		
0–1	133 (53.63)	
2–5	115 (46.37)	
**ECOG**		
0–1	230 (92.74)	
2–5	18 (7.26)	
**B symptom**		
+	79 (31.85)	
−	169 (68.15)	
**Extra nodal sites**		
+	156 (62.90)	
−	92 (37.10)	
**Bone marrow involvement**		
+	21 (8.47)	
−	227 (91.53)	
**HBV infection**		
+	74 (29.84)	
−	174 (70.16)	
**Bulky tumor (>10 cm)**		
+	55 (22.18)	
−	193 (77.82)	
**Elevated LDH**		
+	98 (39.52)	
−	150 (60.48)	
**Elevated β2-MG**		
+	86 (34.68)	
−	162 (65.32)	
**KI-67**		
≤75%	93 (37.50)	
>75%	155 (62.50)	
**Source**		
Gastrointestinal	116 (46.77)	
Others	132 (53.23)	
Response rate		
PR+CR	215 (86.69)	

Abbreviations: CR, complete response; ECOG, Eastern Cooperative Oncology Group; IPI, International Prognostic Index; LDH, lactate dehydrogenase; PR, partial response; β2-MG, β2 macroglobulin.

### Extraction of DNA and genotyping of CREBBP rs3025684

The TIANamp genomic DNA kit (TIANGEN Biotech, Beijing, China) was used to extract the genomic DNA according to the manufacturer’s protocol. Genotypes were analyzed by polymerase chain reaction-restriction fragment length polymorphism (PCR-RFLP). The reaction system contained 60 ng DNA template, 2 μl of each primer, 25 μl Premix Taq (Takara, Dalian, China) and sterilized water up to 50 μl. The PCR protocol was as follows: initial denaturation at 94°C for 5 min, followed by 30 cycles of denaturation at 94°C for 30 s, annealing at 58°C for 30 s, extension at 72°C for 30 s, followed by 72°C for 7 min. The primers for rs3025684 were: forward 5′-AGGGGAAACAACTCACCCTG-3′ and reverse 5′-CTGGTCTTGTGGTTCCGTGT-3′. The PCR product was digested by MnlI (New England Biolabs, Beverly, MA) and analyzed by gel electrophoresis on 2.5% agarose gels. Randomly selected DNA samples were detected by direct sequencing to verify the results (Supplementary Figure S1).

### Isolation of RNA and reverse-transcription quantitative PCR

Total RNA was isolated using TRIzol reagent (Invitrogen). We normalized the levels of expression of *CREBBP* relative to glyceraldehyde phosphate dehydrogenase (GAPDH), and 2^−ΔΔ*C*^_t_ indicated the quantification of gene expression. The primers for *CREBBP* were: forward 5′-CGGCTCTAGTATCAACCCAGG-3′ and reverse 5′- TTTTGTGCTTGCGGATTCAGT-3′. The primers for *GAPDH* were: forward 5′-CCACATCGCTCAGACACCAT-3′ and reverse 5′-CCAGGCGCCCAATACG-3′.

### Statistical analysis

A goodness-of-fit Chi-square test was used to analyze the Hardy–Weinberg equilibrium. The Chi-square test was used to detect the allelic frequency and genotypic distribution in all subjects. We estimated the relationship between *CREBBP* rs3025684 and the susceptibility to DLBCL by unconditional logistic regression. The prognostic factors were explored by univariate Cox regression analysis. The *CREBBP* mRNA expression levels were investigated by the independent Student’s *t* test. The survival curves were computed by Kaplan–Meier method with log-rank tests. All the statistical analyses mentioned above were executed by IBM SPSS Statistics version 20.0. *P*<0.05 was considered to be statistically significant. The false-positive report probability (FPRP) was calculated as previously described [26], we set an FPRP value of 0.2 and assigned a prior probability of 0.1.

## Results

### Characteristics of study subjects

The genotypic distributions in both patients and controls were under the Hardy–Weinberg equilibrium (*P*>0.05). The genotypic distribution and allelic frequencies showed significant difference between the DLBCL patients and healthy individuals for *CREBBP* rs3025684 (*P*=0.021 and *P*=0.013, respectively, shown in Table 2). Furthermore, the association between the genotype and the clinical parameters of DLBCL patients was explored, which showed no statistical differences as shown in Table 3.

**Table 2:** Genotype distribution and allele frequencies of CREBBP rs3025684 polymorphism in DLBCL patients and the controls

	Control (*n*=250) *n* (%)	DLBCL patients (*n*=248) *n* (%)	*P*
Genotype frequency			
GG	153 (61.20)	121 (48.79)	0.021
GA	86 (34.40)	113 (45.56)	
AA	11 (4.40)	14 (5.65)	
Allele frequency			
G	392 (78.40)	355 (71.57)	0.013
A	108 (21.60)	141 (28.43)	

**Table 3:** Characteristics of DLBCL patients and their association with CREBBP rs3025684

Characteristics	Number	Genotype		*P*
	(*n*=248) *n* (%)	GA+AA, *n* (%)	GG, *n* (%)	
**Gender**				
Male	112 (45.16)	61 (24.60)	51 (20.56)	0.352
Female	136 (54.84)	66 (26.61)	70 (28.23)	
**Age**				
≤60	165 (66.53)	87 (35.08)	78 (31.45)	0.500
>60	83 (33.47)	40 (16.13)	43 (17.34)	
**Subtype**				
GCB	75 (30.24)	38 (15.32)	37 (14.92)	0.910
nGCB	173 (69.76)	89 (35.89)	84 (33.87)	
**Ann-arbor stage**				
I–II	152 (61.29)	73 (29.44)	79 (31.85)	0.207
III–IV	96 (38.71)	54 (21.77)	42 (16.94)	
**IPI score**				
0–1	133 (53.63)	62 (25.00)	71 (28.63)	0.120
2–5	115 (46.37)	65 (26.21)	50 (20.16)	
**ECOG**				
0–1	230 (92.74)	114 (45.97)	116 (46.77)	0.064
2–5	18 (7.26)	13 (5.24)	5 (2.02)	
**B symptom**				
+	79 (31.85)	43 (17.34)	36 (14.52)	0.488
−	169 (68.15)	84 (33.87)	85 (34.27)	
**Bone marrow involvement**				
+	21 (8.47)	11 (4.44)	10 (4.03)	0.911
−	227 (91.53)	116 (46.77)	111 (44.76)	
**HBV infection**				
+	74 (29.84)	39 (15.73)	35 (14.11)	0.759
−	174 (70.16)	88 (35.48)	86 (34.68)	
**Bulky tumor (>10 cm)**				
+	55 (22.18)	31 (12.50)	24 (9.68)	0.386
−	193 (77.82)	96 (38.71)	97 (39.11)	
**Elevated LDH**				
+	98 (39.52)	57 (22.98)	41 (16.53)	0.077
−	150 (60.48)	70 (28.23)	80 (32.26)	
**Elevated β2-MG**				
+	86 (34.68)	41 (16.53)	43 (17.34)	0.588
−	162 (65.32)	86 (34.68)	78 (31.45)	
**KI-67**				
≤75%	93 (37.50)	50 (20.16)	43 (17.34)	0.533
>75%	155 (62.50)	77 (31.05)	78 (31.45)	
**Source**				
Gastrointestinal	116 (46.77)	55 (22.18)	61 (24.60)	0.262
Others	132 (53.23)	72 (29.03)	60 (24.19)	

Abbreviations: LDH, lactate dehydrogenase; ECOG, Eastern Cooperative Oncology Group; IPI, International Prognostic Index; β2-MG, β2 macroglobulin.

### Relationship between the *CREBBP* rs3025684 and the susceptibility to DLBCL

The GA genotype was correlated with the risk of DLBCL (odds ratio (OR) = 1.692, 95% confidence interval (95% CI) = 1.170–2.446, *P*=0.005) (Table 4). However, the AA genotype displayed a slightly increased susceptibility to DLBCL with no statistical significance (OR = 1.620, 95% CI = 0.710–3.696, *P*=0.252, *P*=0.252) (Table 4). The combined AA and GA genotype was significantly related to increased susceptibility to DLBCL (OR = 1.684, 95% CI = 1.179–2.404, *P*=0.004) (Table 4). The results for dominant and recessive models are shown in Table 4. As no statistical significance was shown in the dominant model, we did not calculate the FPRP values and statistical power for the dominant model. Positive association was observed in the recessive model and GA genotype as their FPRP value was less than 0.2 (shown in Table 4).

**Table 4:** Association between CREBBP rs3025684 polymorphism and the risk of DLBCL

Genotype	OR (95% CI)	*P*	Statistical power	Prior probability				
				0.25	0.1	0.01	0.001	0.0001
AA vs. GG	1.620 (0.710–3.696)	0.252						
GA vs.GG	1.692 (1.170–2.446)	0.005	0.500	0.030	0.085	0.505	0.0912	0.990
GA/AA vs. GG	1.684 (1.179–2.404)	0.004	0.500	0.024	0.069	0.449	0.891	0.988
GA/GG vs. AA	0.769 (0.342–1.729)	0.526						

### Survival analysis of DLBCL patients according to the *CREBBP* rs3025684

**Figure 1:**
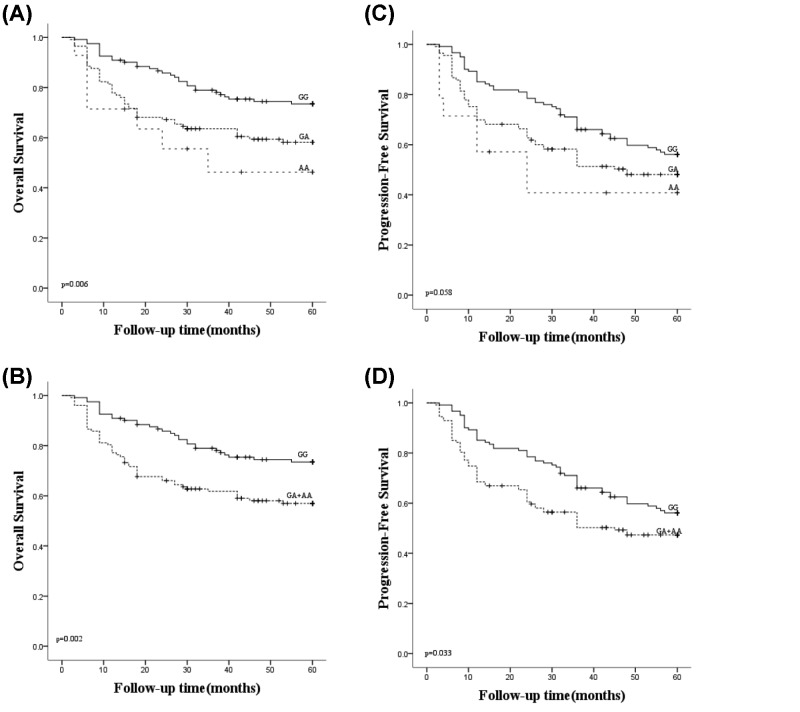
Survival analyses of DLBCL patients under the genotype of *CREBBP* rs3025684 (**A**) OS analysis compared among the GG, GA and AA subgroups (*P*=0.006). (**B**) OS analysis compared between the GG subgroup and the combined GA/AA subgroup (*P*=0.002). (**C**) PFS analysis compared among the GG, GA and AA subgroups (*P*=0.058). (**D**) PFS analysis compared between the GG subgroup and the combined GA/AA subgroup (*P*=0.033). A check mark indicates that the data is censored.

The survival analysis of all 248 DLBCL patients showed that the patients with genotype GA/AA (*P*=0.006, Figure 1A) and the combined GA and AA group (*P*=0.002, Figure 1B) had a worse OS than patients with GG genotype. Furthermore, the patients with GA/AA genotype had a worse progression-free survival (PFS) in comparison with the GG patients with no statistical significance (*P*=0.058, Figure 1C), however, the combined GA and AA group showed worse PFS rate compared with the GG group with statistically significant (*P*=0.033, Figure 1D). Furthermore, it was also indicated that patients with A allele showed poor prognosis (*P*=0.003, HR = 1.944, 95% CI = 1.247–3.029).

### Analysis of the *CREBBP* mRNA expression levels

**Figure 2:**
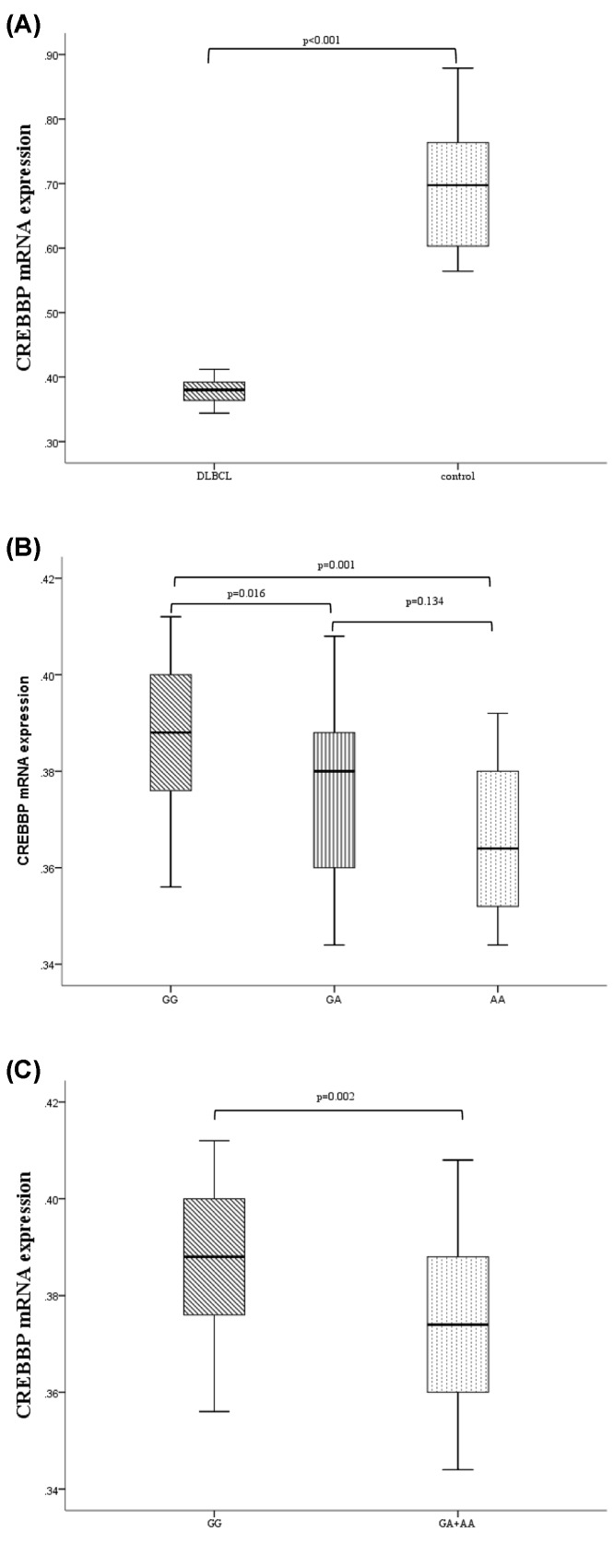
The expression of *CREBBP* mRNA in DLBCL patients (**A**) The expression of *CREBBP* mRNA in DLBCL patients was down-regulated as compared with the controls (*P*<0.001). (**B**) The expression of *CREBBP* mRNA in DLBCL patients with GG genotype was significantly up-regulated than those with GA and AA genotypes (*P*=0.016 and 0.001, respectively). (**C**) The expression of *CREBBP* mRNA in DLBCL patients with GA/AA genotype was significantly down-regulated than those with GG genotype (*P*=0.002).

*CREBBP* expression was detected in 63 patients and 32 controls. The results showed that the CREBBP expression was remarkably down-regulated in patients as compared with controls (*P*<0.001, Figure 2A). The *CREBBP* expression of patients with GG genotype was down-regulated as compared with the patients with GA and AA genotype (*P*=0.016 and 0.001, respectively, Figure 2B). However, no significant difference was detected between the GA and AA subgroups (*P*=0.134, Figure 2B). The *CREBBP* expression was down-regulated in the GA/AA subgroup as compared with the GG subgroup (*P*=0.002, Figure 2C).

### Survival analysis based on the *CREBBP* mRNA levels of DLBCL patients

**Figure 3:**
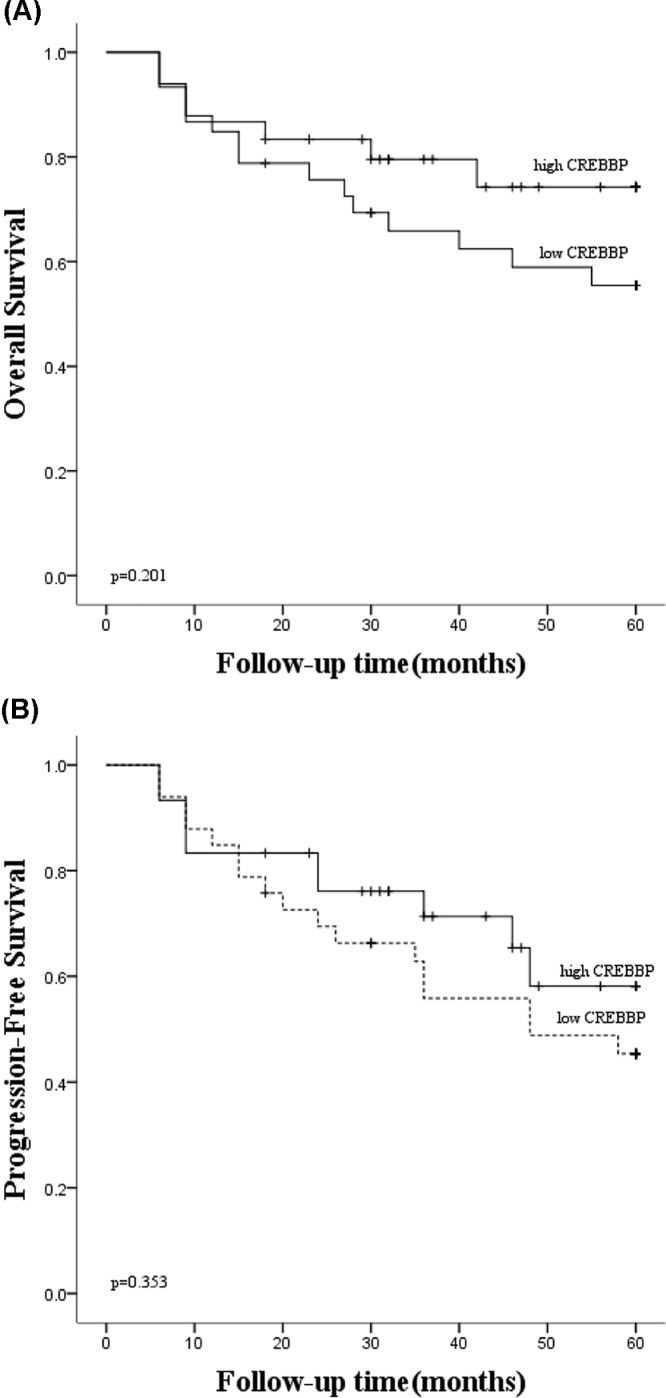
Survival analyses of DLBCL patients according to *CREBBP* mRNA expression (**A**) The OS analysis indicated no significant differences between lower *CREBBP* subgroup and high *CREBBP* subgroup (*P*=0.201). (**B**) The PFS analysis indicated no significant differences between the lower *CREBBP* subgroup and high *CREBBP* subgroup (*P*=0.353). A check mark indicates that the data is censored.

Based on the median of *CREBBP* mRNA expression value, 63 DLBCL patients were partitioned into two subgroups, the low *CREBBP* expression subgroup and the high *CREBBP* expression subgroup. The results indicated no significant differences between the patients with lower *CREBBP* mRNA level and those with high *CREBBP* mRNA level in OS (*P*=0.201, Figure 3A) and PFS (*P*=0.353, Figure 3B).

## Discussion

DLBCL is an example of a translocation-based cancer, in which key genes are dysregulated by active lineage-specific promoters or enhancers due to characteristic equilibrium translocations [6]. The evolution of DLBCL was a multi-step procedure that requires the cumulation of multiple genetic pathological changes [14,27]. By the modern genome-wide molecular analysis, an abundance of altered cellular pathways that perform vital functions in the development of DLBCL and the sensitivity of cancer cells to therapy was detected. However, the notable heterogeneity of this disease partly limits the effective treatment. Therefore, it is necessary to seek ameliorated special biomarkers, permitting the devising of more efficient and accurate medical methods to specific cancer-causing addictions.

*CREBBP*, one of the most frequently mutated genes in DLBCL [15,28–30], acts as a tumor repressor of GC-derived pathogenesis of lymphoma [31]. Most of the *CREBBP-*binding regions in GC B cells displayed characteristics of transcriptionally active (or suspended) enhancers [32], which control the cell type-specific transcription [33]. CREBBP acetylates H3K27 on the promoter or enhancer sequence of the BCL6 target genes, promoting transcription to counteracting the inhibition of BCL6, which leads to the opposition of proto-oncogene activity of BCL6 [20,31]. Therefore, *CREBBP* may be considered as a tumor therapeutic target in DLBCL patients.

Gene expression-based categorization of DLBCL has been established [34,35], and the relevance to prognosis has been illustrated [36]. It has been demonstrated that certain genetic defects and distinct signal transduction pathways occur in specific subtypes [37,38]. In this study, the association between *CREBBP* rs3025684 polymorphism and its expression with the susceptibility and survival of DLBCL patients was investigated. The results showed that the A allele carriers were related to increased risk of DLBCL and had worse OS rate and PFS rate. Hence, *CREBBP* rs3025684 polymorphism may be used as a hallmark for the prediction of risk and prognosis of DLBCL.

In East Asians, the frequency of A allele of CREBBP rs3025684 was reported to be 19.3% in the HapMap Project. However, the present study showed that the frequency of allele A in controls and DLBCL patients was 21.60 and 28.43%, respectively. This difference in allele frequency may owe to the risk effects of allele A and the small sample size. In the recessive model, the positive association between the risk of DLBCL and CREBBP rs3025684 was observed. However, the statistical power was only 0.5, which may be a result of the small simple size.

The CREBBP/EP300 complex participates in numerous life events, such as cell growth, proliferation, apoptosis, metabolism and oncogenesis [18,39,40]. CREBBP/EP300 complex also targets many transcriptional factors significantly related to the development of B-cell lymphoma and immune response, such as p53 and c-MYC [41–43]. HAT activity is related to the survival rate of patients with B-cell lymphoma [31,44]. Furthermore, HAT mutations likely predict treatment efficiency in epigenetically targeted therapy, such as the HDAC3 inhibitors [20,45]. The present study indicated that the expression of *CREBBP* was notably down-regulated in patients as compared with the controls, and was especially down-regulated in the GA/AA genotype subgroup, indicating that CREBBP may be used as a therapeutic target for DLBCL.

In conclusion, the polymorphism and expression of *CREBBP* gene may play a vital role as a genetic risk factor and poor prognostic factor among DLBCL Chinese Han patients, indicating that CREBBP could be used for the prognosis and treatment of DLBCL. Additional studies with larger sample sizes are necessary to verify the results and further functional analyses are warranted to explore the lymphoma biology.

## Supporting information

**Supplementary Figure S1:** 

## Abbreviations

CREBBP: cyclic AMP response element binding protein
DLBCL: diffuse large B-cell lymphoma
FPRP: false-positive report probability
GAPDH: glyceraldehyde phosphate dehydrogenase
HAT: histone acetyltransferase
HDAC3: histone deacetylase 3
HR: hazard ratio
OR: odds ratio
OS: overall survival
PFS: progression-free survival
SNP: single nucleotide polymorphism
95% CI: 95% confidence interval

## References

[1] Lenz G. and Staudt L.M. (2010) Aggressive lymphomas. N. Engl. J. Med. 362, 1417–1429 10.1056/NEJMra0807082 20393178 PMC7316377

[2] Coiffier B., Thieblemont C., Van Den Neste E., Lepeu G., Plantier I., Castaigne S. (2010) Long-term outcome of patients in the LNH-98.5 trial, the first randomized study comparing rituximab-CHOP to standard CHOP chemotherapy in DLBCL patients: a study by the Groupe d’Etudes des Lymphomes de l’Adulte. Blood 116, 2040–2045 10.1182/blood-2010-03-276246 20548096 PMC2951853

[3] Morton L.M., Slager S.L., Cerhan J.R., Wang S.S., Vajdic C.M., Skibola C.F. (2014) Etiologic heterogeneity among non-Hodgkin lymphoma subtypes: the InterLymph Non-Hodgkin Lymphoma Subtypes Project. J. Natl. Cancer Inst. Monogr. 2014, 130–144 10.1093/jncimonographs/lgu013 25174034 PMC4155467

[4] Cerhan J.R. and Slager S.L. (2015) Familial predisposition and genetic risk factors for lymphoma. Blood 126, 2265–2273 10.1182/blood-2015-04-537498 26405224 PMC4643002

[5] Scott D.W. and Gascoyne R.D. (2014) The tumour microenvironment in B cell lymphomas. Nat. Rev. Cancer 14, 517–534 10.1038/nrc3774 25008267

[6] Armitage J.O., Gascoyne R.D., Lunning M.A. and Cavalli F. (2017) Non-Hodgkin lymphoma. Lancet 390, 298–310 10.1016/S0140-6736(16)32407-2 28153383

[7] Abd El-Fattah A.A., Sadik N.A.H., Shaker O.G. and Mohamed Kamal A. (2018) Single nucleotide polymorphism in SMAD7 and CHI3L1 and colorectal cancer risk. Mediators Inflamm. 2018, 9853192 10.1155/2018/9853192 30498395 PMC6222239

[8] Wen J., Lv Z., Ding H., Fang X. and Sun M. (2018) Association of miRNA biosynthesis genes DROSHA and DGCR8 polymorphisms with cancer susceptibility: a systematic review and meta-analysis. Biosci. Rep. 38, 10.1042/BSR20180072 PMC6019356 29654164

[9] Liu C., Cui H., Gu D., Zhang M., Fang Y., Chen S. (2017) Genetic polymorphisms and lung cancer risk: Evidence from meta-analyses and genome-wide association studies. Lung Cancer 113, 18–29 10.1016/j.lungcan.2017.08.026 29110844

[10] Liu G.C., Zhou Y.F., Su X.C. and Zhang J. (2019) Interaction between TP53 and XRCC1 increases susceptibility to cervical cancer development: a case control study. BMC Cancer 19, 24 10.1186/s12885-018-5149-0 30616520 PMC6323714

[11] Li Y., Bai O., Cui J. and Li W. (2016) Genetic polymorphisms in the DNA repair gene, XRCC1 associate with non-Hodgkin lymphoma susceptibility: a systematic review and meta-analysis. Eur. J. Med. Genet. 59, 91–103 10.1016/j.ejmg.2015.12.011 26723520

[12] Wenzl K., Hofer S., Troppan K., Lassnig M., Steinbauer E., Wiltgen M. (2016) Higher incidence of the SNP Met 788 Ile in the coding region of A20 in diffuse large B cell lymphomas. Tumour Biol. 37, 4785–4789 10.1007/s13277-015-4322-1 26518771

[13] Liu Y., Wang X., Ding N., Mi L., Ping L., Jin X. (2017) TP53 Arg72 as a favorable prognostic factor for Chinese diffuse large B-cell lymphoma patients treated with CHOP. BMC Cancer 17, 743 10.1186/s12885-017-3760-0 29126407 PMC5680759

[14] Cerhan J.R., Berndt S.I., Vijai J., Ghesquieres H., McKay J., Wang S.S. (2014) Genome-wide association study identifies multiple susceptibility loci for diffuse large B cell lymphoma. Nat. Genet. 46, 1233–1238 10.1038/ng.3105 25261932 PMC4213349

[15] Pasqualucci L., Dominguez-Sola D., Chiarenza A., Fabbri G., Grunn A., Trifonov V. (2011) Inactivating mutations of acetyltransferase genes in B-cell lymphoma. Nature 471, 189–195 10.1038/nature09730 21390126 PMC3271441

[16] Bartsch O., Schmidt S., Richter M., Morlot S., Seemanova E., Wiebe G. (2005) DNA sequencing of CREBBP demonstrates mutations in 56% of patients with Rubinstein-Taybi syndrome (RSTS) and in another patient with incomplete RSTS. Hum. Genet. 117, 485–493 10.1007/s00439-005-1331-y 16021471

[17] Stef M., Simon D., Mardirossian B., Delrue M.A., Burgelin I., Hubert C. (2007) Spectrum of CREBBP gene dosage anomalies in Rubinstein-Taybi syndrome patients. Eur. J. Hum. Genet. 15, 843–847 10.1038/sj.ejhg.5201847 17473832

[18] Goodman R.H. and Smolik S. (2000) CBP/p300 in cell growth, transformation, and development. Genes Dev. 14, 1553–1577 10887150

[19] Kalkhoven E. (2004) CBP and p300: HATs for different occasions. Biochem. Pharmacol. 68, 1145–1155 10.1016/j.bcp.2004.03.045 15313412

[20] Jiang Y., Ortega-Molina A., Geng H., Ying H.Y., Hatzi K., Parsa S. (2017) CREBBP inactivation promotes the development of HDAC3-dependent lymphomas. Cancer Discov. 7, 38–53 10.1158/2159-8290.CD-16-0975 27733359 PMC5300005

[21] Gao C., Zhang R.D., Liu S.G., Zhao X.X., Cui L., Yue Z.X. (2017) Low CREBBP expression is associated with adverse long-term outcomes in paediatric acute lymphoblastic leukaemia. Eur. J. Haematol. 99, 150–159 10.1111/ejh.12897 28452416

[22] Kung A.L., Rebel V.I., Bronson R.T., Ch’ng L.E., Sieff C.A., Livingston D.M. (2000) Gene dose-dependent control of hematopoiesis and hematologic tumor suppression by CBP. Genes Dev. 14, 272–277 10673499 PMC316359

[23] Barnby G., Abbott A., Sykes N., Morris A., Weeks D.E., Mott R. (2005) Candidate-gene screening and association analysis at the autism-susceptibility locus on chromosome 16p: evidence of association at GRIN2A and ABAT. Am. J. Hum. Genet. 76, 950–966 10.1086/430454 15830322 PMC1196454

[24] Kumar D., Deb I., Chakraborty J., Mukhopadhyay S. and Das S. (2011) A polymorphism of the CREB binding protein (CREBBP) gene is a risk factor for addiction. Brain Res. 1406, 59–64 10.1016/j.brainres.2011.05.048 21752352

[25] Turner J.J., Hughes A.M., Kricker A., Milliken S., Grulich A., Kaldor J. (2005) WHO non-Hodgkin’s lymphoma classification by criterion-based report review followed by targeted pathology review: an effective strategy for epidemiology studies. Cancer Epidemiol. Biomarkers Prev. 14, 2213–2219 10.1158/1055-9965.EPI-05-0358 16172234

[26] Wacholder S., Chanock S., Garcia-Closas M., El Ghormli L. and Rothman N. (2004) Assessing the probability that a positive report is false: an approach for molecular epidemiology studies. J. Natl. Cancer Inst. 96, 434–442 10.1093/jnci/djh075 15026468 PMC7713993

[27] Schmitz R., Wright G.W., Huang D.W., Johnson C.A., Phelan J.D., Wang J.Q. (2018) Genetics and pathogenesis of diffuse large B-cell lymphoma. N. Engl. J. Med. 378, 1396–1407 10.1056/NEJMoa1801445 29641966 PMC6010183

[28] Lohr J.G., Stojanov P., Lawrence M.S., Auclair D., Chapuy B., Sougnez C. (2012) Discovery and prioritization of somatic mutations in diffuse large B-cell lymphoma (DLBCL) by whole-exome sequencing. Proc. Natl. Acad. Sci. U.S.A. 109, 3879–3884 10.1073/pnas.1121343109 22343534 PMC3309757

[29] Morin R.D., Mendez-Lago M., Mungall A.J., Goya R., Mungall K.L., Corbett R.D. (2011) Frequent mutation of histone-modifying genes in non-Hodgkin lymphoma. Nature 476, 298–303 10.1038/nature10351 21796119 PMC3210554

[30] Pasqualucci L., Trifonov V., Fabbri G., Ma J., Rossi D., Chiarenza A. (2011) Analysis of the coding genome of diffuse large B-cell lymphoma. Nat. Genet. 43, 830–837 10.1038/ng.892 21804550 PMC3297422

[31] Zhang J., Vlasevska S., Wells V.A., Nataraj S., Holmes A.B., Duval R. (2017) The CREBBP acetyltransferase is a haploinsufficient tumor suppressor in B-cell lymphoma. Cancer Discov. 7, 322–337 10.1158/2159-8290.CD-16-1417 28069569 PMC5386396

[32] Heinz S., Romanoski C.E., Benner C. and Glass C.K. (2015) The selection and function of cell type-specific enhancers. Nat. Rev. Mol. Cell Biol. 16, 144–154 10.1038/nrm3949 25650801 PMC4517609

[33] Hnisz D., Abraham B.J., Lee T.I., Lau A., Saint-Andre V., Sigova A.A. (2013) Super-enhancers in the control of cell identity and disease. Cell 155, 934–947 10.1016/j.cell.2013.09.053 24119843 PMC3841062

[34] Alizadeh A.A., Eisen M.B., Davis R.E., Ma C., Lossos I.S., Rosenwald A. (2000) Distinct types of diffuse large B-cell lymphoma identified by gene expression profiling. Nature 403, 503–511 10.1038/35000501 10676951

[35] Monti S., Savage K.J., Kutok J.L., Feuerhake F., Kurtin P., Mihm M. (2005) Molecular profiling of diffuse large B-cell lymphoma identifies robust subtypes including one characterized by host inflammatory response. Blood 105, 1851–1861 10.1182/blood-2004-07-2947 15550490

[36] Lenz G., Wright G., Dave S.S., Xiao W., Powell J., Zhao H. (2008) Stromal gene signatures in large-B-cell lymphomas. N. Engl. J. Med. 359, 2313–2323 10.1056/NEJMoa0802885 19038878 PMC9103713

[37] Davis R.E., Ngo V.N., Lenz G., Tolar P., Young R.M., Romesser P.B. (2010) Chronic active B-cell-receptor signalling in diffuse large B-cell lymphoma. Nature 463, 88–92 10.1038/nature08638 20054396 PMC2845535

[38] Ngo V.N., Young R.M., Schmitz R., Jhavar S., Xiao W., Lim K.H. (2011) Oncogenically active MYD88 mutations in human lymphoma. Nature 470, 115–119 10.1038/nature09671 21179087 PMC5024568

[39] Giordano A. and Avantaggiati M.L. (1999) p300 and CBP: partners for life and death. J. Cell. Physiol. 181, 218–230 10.1002/(SICI)1097-4652(199911)181:2<218::AID-JCP4>3.0.CO;2-5 10497301

[40] Giles R.H., Peters D.J. and Breuning M.H. (1998) Conjunction dysfunction: CBP/p300 in human disease. Trends Genet. 14, 178–183 10.1016/S0168-9525(98)01438-3 9613201

[41] Gu W. and Roeder R.G. (1997) Activation of p53 sequence-specific DNA binding by acetylation of the p53 C-terminal domain. Cell 90, 595–606 10.1016/S0092-8674(00)80521-8 9288740

[42] Vervoorts J., Luscher-Firzlaff J.M., Rottmann S., Lilischkis R., Walsemann G., Dohmann K. (2003) Stimulation of c-MYC transcriptional activity and acetylation by recruitment of the cofactor CBP. EMBO Rep. 4, 484–490 10.1038/sj.embor.embor821 12776737 PMC1319176

[43] Weaver B.K., Kumar K.P. and Reich N.C. (1998) Interferon regulatory factor 3 and CREB-binding protein/p300 are subunits of double-stranded RNA-activated transcription factor DRAF1. Mol. Cell. Biol. 18, 1359–1368 10.1128/MCB.18.3.1359 9488451 PMC108849

[44] Juskevicius D., Jucker D., Klingbiel D., Mamot C., Dirnhofer S. and Tzankov A. (2017) Mutations of CREBBP and SOCS1 are independent prognostic factors in diffuse large B cell lymphoma: mutational analysis of the SAKK 38/07 prospective clinical trial cohort. J. Hematol. Oncol. 10, 70 10.1186/s13045-017-0438-7 28302137 PMC5356266

[45] Andersen C.L., Asmar F., Klausen T., Hasselbalch H. and Gronbaek K. (2012) Somatic mutations of the CREBBP and EP300 genes affect response to histone deacetylase inhibition in malignant DLBCL clones. Leukemia Res. Rep. 2, 1–3 24371765 10.1016/j.lrr.2012.10.002 PMC3850379

