# Supplementary figures and images for: Genetic polymorphism and transcriptional regulation of CREBBP gene in patient with diffuse large B-cell lymphoma
Source: Biosci Rep. 2019 Aug 13;39(8):BSR20191162. doi: 10.1042/BSR20191162 (PMC6692565; doi:10.1042/BSR20191162)

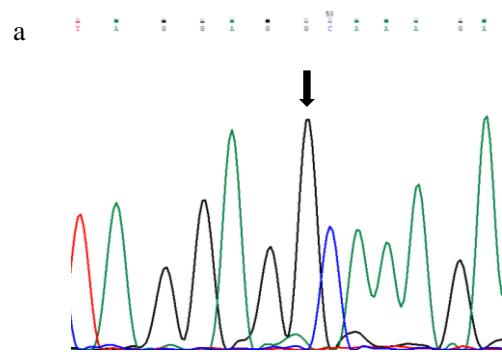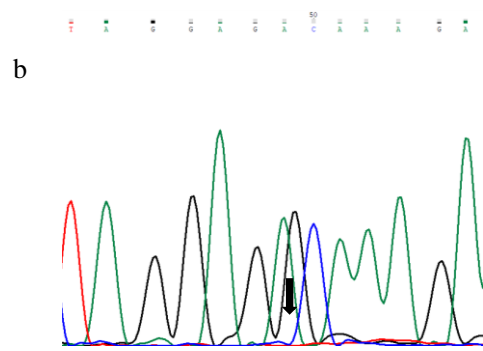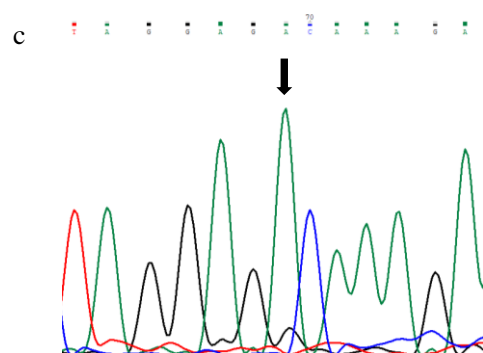

Supplement: Supplementary file 1 [file bsr20191162_Supp1.pdf]
